# Bacterial diversity in the water column of meromictic Lake Cadagno and evidence for seasonal dynamics

**DOI:** 10.1371/journal.pone.0209743

**Published:** 2018-12-26

**Authors:** Francesco Danza, Damiana Ravasi, Nicola Storelli, Samuele Roman, Samuel Lüdin, Matthieu Bueche, Mauro Tonolla

**Affiliations:** 1 Laboratory of Applied Microbiology (LMA), Department for Environmental Constructions and Design (DACD), University of Applied Sciences and Arts of Southern Switzerland (SUPSI), Bellinzona, Switzerland; 2 Microbiology Unit, Department of Botany and Plant Biology, University of Geneva, Geneva, Switzerland; 3 Alpine Biology Center Foundation, Bellinzona, Switzerland; 4 Federal Office for Civil Protection, Spiez Laboratory, Biology Division, Spiez, Switzerland; 5 Institute of Chemistry, University of Neuchâtel, Neuchâtel, Switzerland; 6 Bueche μlab, Le Pâquier, Switzerland; Free University of Bozen/Bolzano, ITALY

## Abstract

The meromictic Lake Cadagno is characterized by a compact chemocline with high concentrations of anoxygenic phototrophic purple and green sulfur bacteria. However, a complete picture of the bacterial diversity, and in particular of effects of seasonality and compartmentalization is missing. To characterize bacterial communities and elucidate relationships between them and their surrounding environment high-throughput 16S rRNA gene pyrosequencing was conducted. Proteobacteria, Chlorobi, Verrucomicrobia, and Actinobacteria were the dominant groups in Lake Cadagno water column. Moreover, bacterial interaction within the chemocline and between oxic and anoxic lake compartments were investigated through fluorescence *in situ* hybridization (FISH) and flow cytometry (FCM). The different populations of purple sulfur bacteria (PSB) and green sulfur bacteria (GSB) in the chemocline indicate seasonal dynamics of phototrophic sulfur bacteria composition. Interestingly, an exceptional bloom of a cyanobacteria population in the oxic-anoxic transition zone affected the common spatial distribution of phototrophic sulfur bacteria with consequence on chemocline location and water column stability. Our study suggests that both bacterial interactions between different lake compartments and within the chemocline can be a dynamic process influencing the stratification structure of Lake Cadagno water column.

## Introduction

Composition and diversity of bacterial communities in lakes are determined by environmental factors with geochemistry playing a dominant role. In turn, community composition and inter-specific interactions determine ecosystem functioning [1]. Globally, microbial photosynthetic carbon fixation links the carbon cycle with the cycles of sulfur (chemolithotrophic sulfur oxidizers and anoxygenic photosynthetic bacteria), nitrogen (nitrifiers and ANAMMOX bacteria) and iron (photoferrotrophs and chemolithoautotrophs) [2–4]. Moreover, bacterial metabolic activity relates directly to biogeochemical cycles of these and other elements, as different electron acceptors can be used for the respiration of organic matter. Knowledge of the principal factors controlling composition and diversity of bacterial communities in lake ecosystems is thus crucial to understand consequences on biogeochemical processes and ecosystem functioning under current environmental changes, considering that global warming influences bacterial population size and diversity [5–10].

Among aquatic environments, meromictic lakes are ideal ecosystems to study bacterial diversity in confined and stratified systems [11]. The oxic and anoxic layers are permanently separated by a chemocline and create a steep redox gradient, resulting in stratification of bacterial metabolic processes. Strong gradients of salinity, dissolved gases (such as oxygen and sulfide), and nutrients in the chemocline offer habitats for functionally distinct bacterial communities [12–15]. In particular, light and sulfide gradients may favor the development of an important community of anaerobic phototrophic sulfur bacteria [11,16].

Lake Cadagno (maximal depth 21 m) is a crenogenic meromictic lake located in the southern slopes of the Swiss Alps. The lake is permanently stratified with a chemocline between 10 and 14 m depth [17,18]. Sulfide gradients in the chemocline foster the development of a particular bacterial community dominated by phototrophic sulfur bacteria, mainly purple sulfur bacteria (PSB) of the family Chromatiaceae (phylum Proteobacteria) [17,19], and green sulfur bacteria (GSB) of the family Chlorobiaceae (phylum Chlorobi) [20,21]. Earlier studies have investigated the evolution of the phototrophic microbial community in the chemocline of Lake Cadagno [20–23]. They revealed that before the year 2000, the bacterial community was dominated by anoxygenic phototrophic PSB of the genera *Chromatium*, *Lamprocystis*, *Thiocystis* and *Thiodictyon* [17–19] representing up to 80% of the phototrophic sulfur bacteria community [20]. In contrast, GSB were present at relatively low abundances with the species *Chlorobium phaeobacteroides* (< 20% of phototrophic sulfur bacteria). After the year 2001, a clonal population of GSB *Chlorobium clathratiforme*, a previously undetected species, became dominant and produced a shift in dominance from PSB to GSB [20,21]. At the same time, the total abundance of phototrophic sulfur bacteria increased from approximately 10^6^ to 10^7^ cells mL^-1^ [20,21]. Nevertheless, the dominance of GSB did not result in detectable changes in the distribution and abundance of PSB populations [20]. These marked changes in community composition of phototrophic sulfur bacteria are likely to affect ecosystem processes. However, the rate of inorganic carbon fixation of the novel *C*. *clathratiforme* population was very low compared to PSB *Chromatium okenii* and *Candidatus* “Thiodictyon syntrophicum” strain Cad16^T^ [24–26].

The coexistence of phylogenetically distinct bacteria with substantial metabolic redundancy such as PSB and GSB might be relevant for maintenance of key ecosystem functions in Lake Cadagno. However, yet unknown differences in other functional characteristics of the species involved are required to explain their coexistence [27]. Although anoxygenic phototrophic sulfur bacteria are confined to the chemocline, they interact with the bacterial community in adjacent strata of the water column [18]. For instance, strong interactions have been shown with sulfate-reducing bacteria (SRB) in the chemocline and the anoxic monimolimnion [28,29]. Furthermore, methane oxidation in the chemocline is performed by gamma-proteobacterial aerobic methane-oxidizers active in the anoxic waters coupled to *in situ* oxygen production by photosynthetic plankton, mainly cyanobacteria [30,31]. Interactions among distinct bacterial taxa and the composition of the bacterial community in the interlinked strata of the water column thus seem important determinants of ecosystem functioning [32]. These interactions are yet unknown for Lake Cadagno and similar meromictic lakes, whose functioning relies strongly on the bacterial community.

Our study aimed at providing a complete picture of bacterial diversity in the water column of Lake Cadagno and at elucidating the role of bacterial interactions within and between its different strata. To this end, we characterized the bacterial community of the entire water column using high-throughput 454 sequencing and investigated possible bacterial interactions between lake compartments and within the chemocline and their consequences for the whole ecosystem. In particular, seasonal dynamics of PSB and GSB community composition in the chemocline were determined through fluorescence *in situ* hybridization (FISH). Furthermore, we used flow cytometry (FCM) to monitor interactions between phototrophic sulfur bacteria in the chemocline and with cyanobacteria at the interface of the oxic and anoxic compartments.

## Material and methods

### Study site and sampling procedures

Lake Cadagno is a permanently stratified (meromictic) lake located in the southern Swiss Alps at 1921 m above sea level with a maximum depth of 21 m (46.55087°N, 8.71152°E). In addition to surface water tributaries, the lake is fed by underwater springs passing through gypsum-rich dolomite rock and transporting salts, including sulfate, to the bottom water. Water from these springs create an anaerobic, sulfidic monimolimnion with high salinity and an aerobic, low-salinity mixolimnion, both separated by a chemocline at 10–14 m depth [17,18].

Sampling of Lake Cadagno for high-throughput 454 sequencing was performed in October 2012 using a 1 L Ruttner sampler (Hydrobios Apparatebau GmbH Altenholz–Germany). One liter of water was collected from several depths throughout the entire water column: i.e. from 2, 4, 6, 8, 11.5, 14, 16 and 18 m.

Sampling of Lake Cadagno water column for FCM was performed in July 2016, July 2017 and August 2017. Sampling for FISH analyses was performed in July 2016 and October 2016 using the same procedure described above.

All samples were kept in dark and cold conditions until transport to the laboratory, where they were processed within one hour.

The University of Geneva has an official permission from the Government of Canton Ticino (Switzerland) for the scientific works on Lake Cadagno.

### Physico-chemical parameters of Lake Cadagno

Turbidity (NTU) in the water column was measured with a turbidity sensor (ECO NTU, WET Labs, Sea-Bird, Bellevue, WA, USA) attached to a Sea-Bird CTD (conductivity, temperature, depth; SBE 19plus V2, Sea-Bird, Bellevue, WA, USA). The sensor measured backscattered light emitted at 700 nm with a sensitivity of 0.02 NTU. Oxygen (mg L^-1^) was measured with a membrane-based probe (OxyGuard, Ocean Probe, Farum, Denmark) mounted on another multiparameter probe (CTM281, Sea & Sun Technology, Trappenkamp, Germany) measuring at 2.4Hz, which was lowered together with the CTD. For the sulfide analysis, 12 mL subsamples were immediately transferred to screw capped tubes containing 0.8 mL of 4% zinc acetate solution. These solutions were stored in the dark and analyzed colorimetrically using a Spectroquant kit (Merck, Schaffhausen, Switzerland).

### DNA extraction, amplicon library generation and pyrosequencing

Water samples were filtered onto 0.2 μm cellulose-nitrate membrane filters (Sartorius Stedim Biotech, Goettingen, Germany). Each filter was then inserted into a small plastic envelope. Three mL of TE (Tris-EDTA) buffer were added and bacterial cells were resuspended by manually rubbing the filter. The supernatant containing the bacterial cells was then transferred into another envelope. This procedure resulted in samples of concentrated microbial cells in TE buffer solution from the three layers of Lake Cadagno water column.

Total DNA was then extracted from the supernatant through proteinase K and extraction protocol based on phenol/chloroform optimized for the extraction of DNA from bacterial plankton [33]. DNA concentrations ranged from 77 to 149 ng/μL. For each sample, around 1000 ng of DNA was sent to an external laboratory (Research and Testing Laboratory (LCC) in Lubbock, Texas, USA) for pyrosequencing (454 GS FLX Titanium platform; 454 Life Sciences, Roche, Branford, USA). The primers used for the amplification of 16S rRNA genes were 28F (5’-GAG TTT GAT CNT GGC TCA G-3’) and 519R (5-GWA TTA CCG CGG CKG CTG-3’) allowing the recovery of short fragments of approximately 400 bp in the 16S rRNA gene that included the V1-V3 hypervariable regions.

### Sequence and data analysis

Starting from FastA and FastQ output files, data were analysed using the Qiime software distributed as Virtual machine version 13_8 [34]. In short, data were first filtered to remove too short and too long reads and reads low quality. The data were then demultiplexed based on a barcode identifier, allowing the recovery of the sequences corresponding to the three different water samples (i.e. mixolimnion, chemocline, monimolimnion). Operational taxonomic units (OTUs) were then defined (97% sequence identity) and identified using the “de novo OTU picking tool” from Qiime, which is a multi-step process. For every OTU, the taxonomical assignation was performed on a representative sequence using UCLUST [35] and the Greengenes reference database. Qiime was also used to compute rarefaction curves based on CHAO1 metrics. The relative abundance of bacterial communities at various taxonomic levels was determined and Shannon-Weaver diversity index calculated.

### Flow cytometry analysis

For the detection of autofluorescent phototrophic bacteria in the water column, FCM was used to determine chlorophyll/bacteriochlorophyll and phycobilin signatures as described in previous studies [27,36,37]. We used a BD Accuri C6 cytometer (Becton Dickinson, San José, CA, USA) device equipped with two lasers (488 nm, 680 nm), two scatter detectors, and four fluorescence detectors (laser 488nm: FL1 = 533/30, FL2 = 585/40, FL3 = 670; laser 640 nm: FL4 = 670). Two parameters were used for event characterization: forward scatter (FSC) which correlates to particle size, and 90° light scatter (SSC), correlating to internal granularity of the particles.

For the identification of phototrophic bacteria, a first forward scatter threshold of FSC-H 10’000 was applied to exclude debris and abiotic particles. Subsequently a FL3-A > 1’100 threshold was applied using FL3 (red fluorescence), to select cells emitting autofluorescence due to chlorophyll and bacteriochlorophyll. For cyanobacteria of the oxic-anoxic zone, the 640-nm red laser was used to excite phycocyanins in the light-harvesting phycobilisomes with emissions detected in FL4 (675 ± 12.5 nm). A FL4-A > 1’100 was applied to select cells emitting autofluorescence due to phycocyanin. Sample analysis was limited to 50 μL with fluidic flow rate of 66 μL min^-1^ and samples were diluted if necessary in order to achieve a maximum of 1’000 events per mL. Green sulfur (GSB) and purple sulfur bacteria (PSB) colonizing the chemocline of Lake Cadagno were distinguished through FCM based on morphological characters as described by Danza et al [27]. Among PSB, large-celled *C*. *okenii* (~ 7 μm) and GSB *Chlorobium* spp. (~ 0.8 μm) were clearly separated from the other populations in SSC vs FSC dot-plots. PSB *C*. *okenii*, GSB *Chlorobium* and cyanobacteria gating permitted their respective counts.

### Fluorescence in situ hybridization

Anoxygenic PSB and GSB were identified and quantified using fluorescent *in situ* hybridization (FISH) with species- specific Cy3-labeled oligonucleotides (Table 1) in 1-μL aliquots of paraformaldehyde-fixed water samples (n = 3) spotted onto gelatin-coated slides (0.1% gelatin, 0.01% KCr(SO_4_)_2_) [38]. Hybridizations were performed as described by [39] with concomitant DAPI staining for total bacterial community quantification. The slides were treated with Citifluor AF1 (Citifluor Ltd., London, UK) and examined by epifluorescence microscopy using filter sets F31 (AHF Analysentechnik, Tübingen, Germany; D360/40, 400DCLP, and D460/50 for DAPI) and F41 (AHF Analysentechnik; HQ535/50, Q565LP, and HQ610/75 for Cy3). The microorganisms were counted at 1000 × magnification in 40 fields of 0.01 mm^2^ [40].

**Table 1:** Cy3-labeled oligonucleotide probes used in this study for FISH counting.

Probe	Target	Sequence (5’ → 3’) (formamide concentration in hybridization buffer)	Reference
GAM42a	γ-subdivision of proteobacteria	GCCTTCCCACATCGTTT (30%)	[41]
CMOK	*Chromatium okenii*	AGCCGATGGGTATTAACCACCAGGTT (30%)	[19]
S453D	clone 2/61 γ-subdivision of proteobacteria	CAGCCCAGGGTATTAACCCAAGCCGC (40%)	[19]
S453F	“Thiodictyon syntrophicum” strain Cad16^T^	CCCTCATGGGTATTARCCACAAGGCG (40%)	[19]
S453E	*Lamprocystis roseopersicina*	CATTCCAGGGTATTAACCCAAAATGC (30%)	[19]
S453A	*Lamprocystis purpurea*	TCGCCCAGGGTATTATCCCAAACGAC (40%)	[19]
GSB	Green sulfur bacteria, *Chlorobiaceae*	GGCAGAACAACCATGCGATTGT	[20]

## Results

### Physico-chemical analysis of Lake Cadagno water column

**Fig 1:**
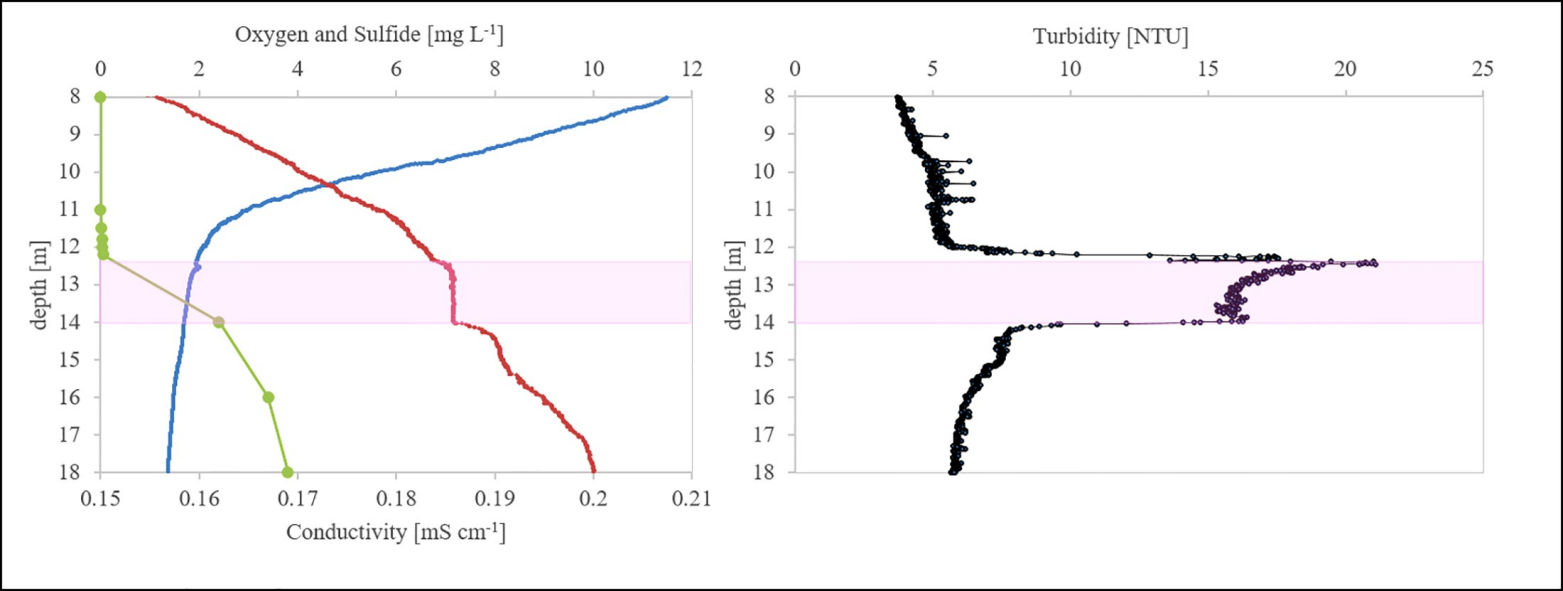
Physico-chemical and turbidity profiles of Lake Cadagno water column. Oxygen [mg L^-1^, blue line], H_2_S [mg L^-1^], conductivity [mS cm^-1^] (left), and turbidity profile [NTU] (right) (12 July 2016). The pink shadow highlight the population of anoxygenic phototrophic sulfur bacteria.

Physico-chemical parameter profiles confirmed the typical stratification of Lake Cadagno (Fig 1), with the chemocline at 12–14 m characterized by a turbidity maximum, a homogeneous conductivity zone and a sharp decline of oxygen and a concomitant increase of sulfide with increasing depth. (S1 Fig displays physico-chemical parameters for successive sampling dates).

### High-throughput sequencing of bacterial community in Lake Cadagno

After quality filtering, a total of 9’039 sequences (2’053 in the mixolimnion, 2’610 in the chemocline, and 4’376 in the monimolimnion) were retained (average sequence length of 398 bp), which clustered into 504 OTUs. For all the samples, rarefaction curves based on CHAO1 metrics reached the plateau (S2 Fig), indicating that sequencing effort was sufficient to characterize the communities from the three compartments. All retained sequences belonged to Bacteria and comprised 15 phyla, 40 classes and 115 genera. The greatest bacterial diversity was observed in anoxic chemocline and monimolimnion with Shannon-Weaver values of 5.44 and 5.27, respectively, whereas in the mixolimnion Shannon-Weaver value was 4.49.

**Fig 2:**
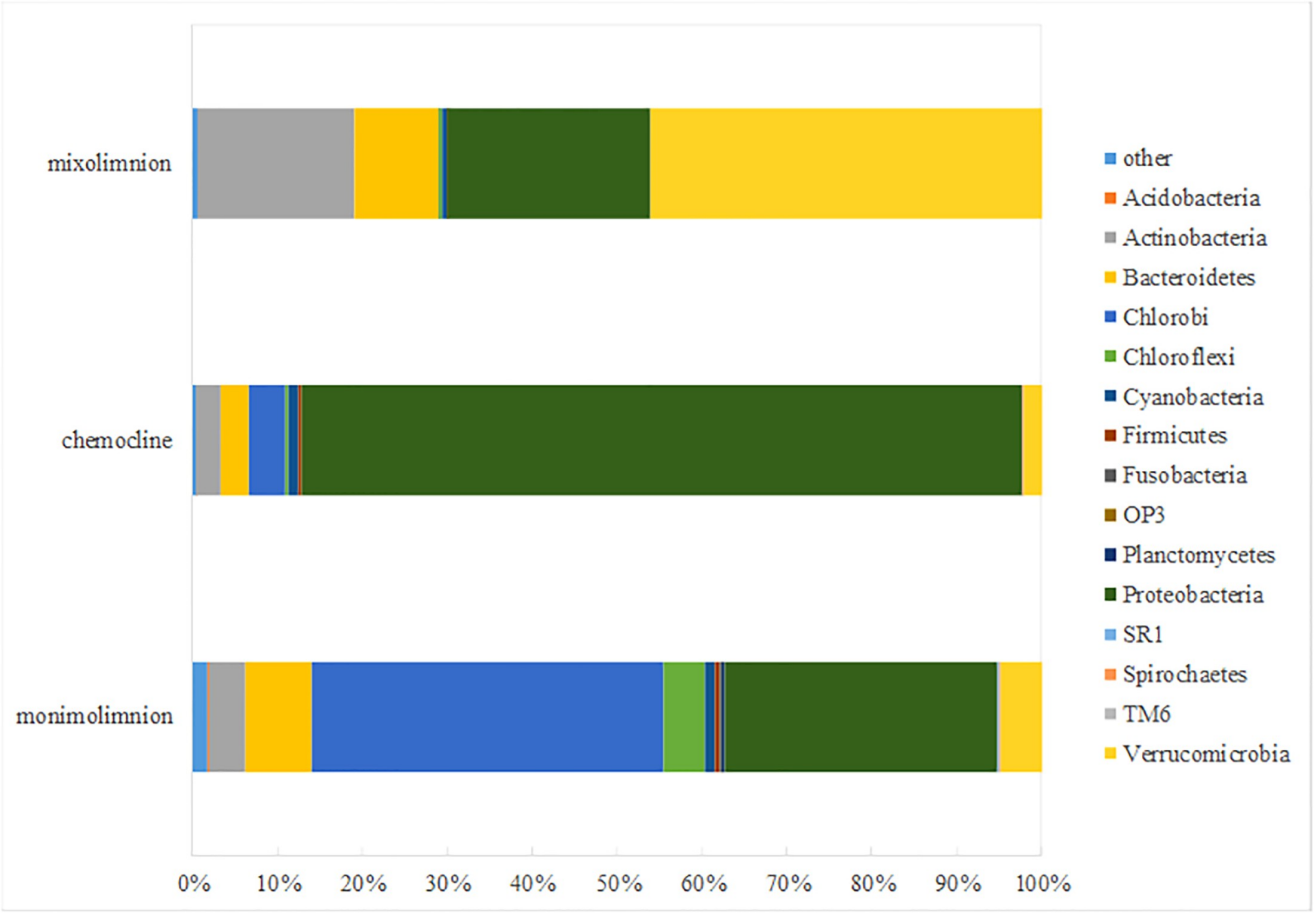
Percentage of relative abundance of bacterial communities at the phylum level according to mixolimnion, chemocline and monimolimnion water layers in Lake Cadagno (October 2012).

At the phylum level, Proteobacteria, Chlorobi, Verrucomicrobia, and Actinobacteria were the dominant groups in Lake Cadagno (Fig 2). In the mixolimnion only, Verrucomicrobia (46% relative abundance) and Proteobacteria (23%) were the major bacterial phyla. In the chemocline, Proteobacteria (84%) dominated, whereas Cyanobacteria were present at 1% total abundance. In the monimolimnion, Chlorobi (41%) and Proteobacteria (32%) were the dominant phyla. The Proteobacteria phylum was present in the entire water column, and its relative abundance and distribution at the class level was dominated by Alphaproteobacteria in the mixolimnion (75% of Proteobacteria abundance), whereas Gammaproteobacteria dominated the chemocline (95% of Proteobacteria abundance) and the monimolimnion (67% of Proteobacteria abundance). Deltaproteobacteria and Epsilonproteobacteria were also present in the monimolimnion, both at around 11% of total Proteobacteria abundance.

**Fig 3:**
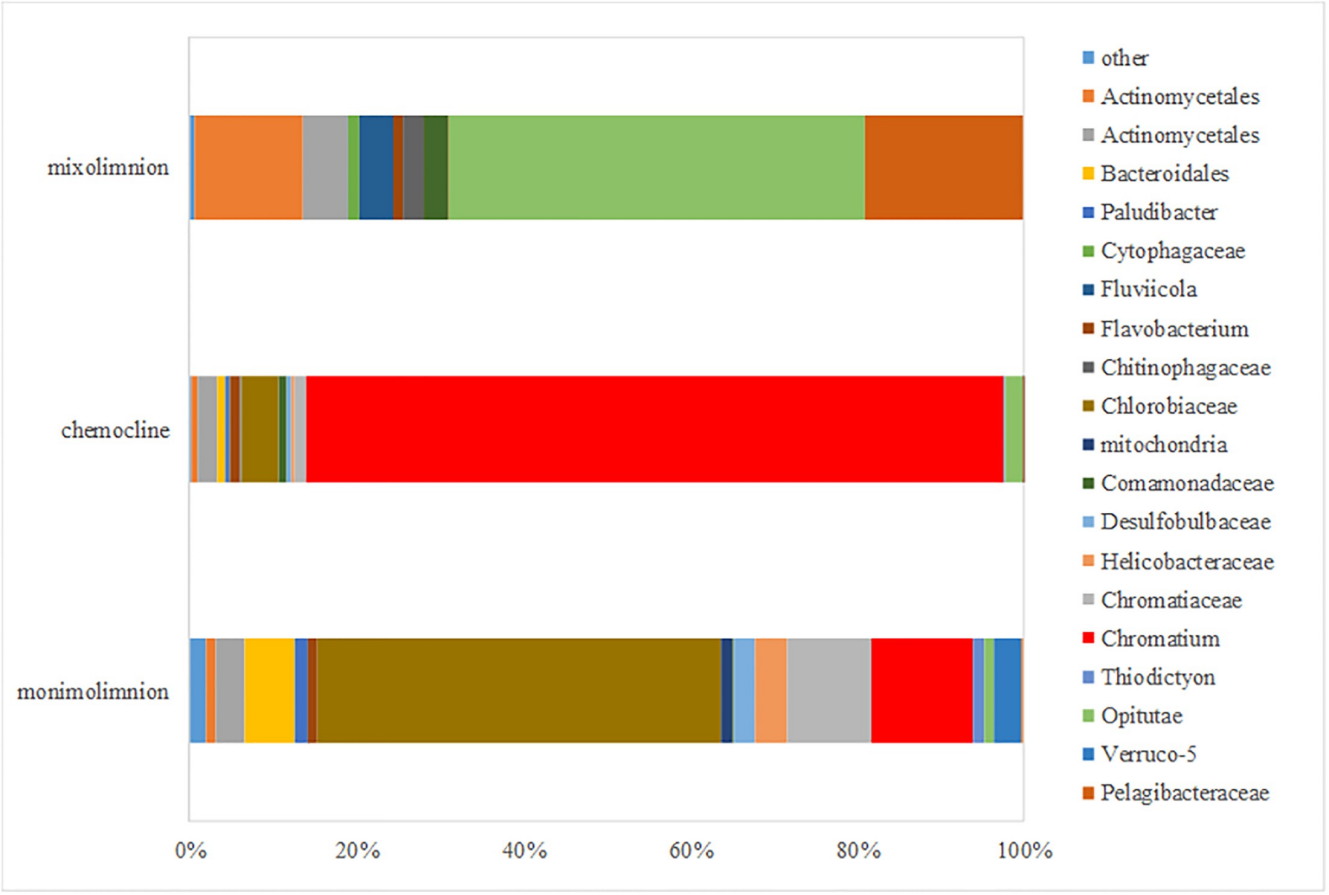
Percentage of relative abundance of bacterial communities at the family and genus level according to mixolimnion, chemocline and monimolimnion water layers in Lake Cadagno (October 2012).

Total relative abundances and distributions of bacterial families and genera across the main water layers were as follows (Fig 3). The mixolimnion community was dominated by the genus *Opitutus* (45% of total abundance, belonging to Verrucomicrobia phylum) and the family Pelagibacteraceae (17%, belonging to Proteobacteria phylum). *Chromatium* dominated in the chemocline (78%), followed by Chlorobiaceae (4%). In the monimolimnion, Chlorobiaceae amounted to 41% of total abundance, whereas *Chromatium* represented 10% of total abundance. Genera within Desulfobulbaceae (2%, belonging to Deltaproteobacteria) and Helicobacteraceae (3%, Epsilonproteobacteria) were also detected in the monimolimnion.

### Fine-scale analysis of anoxygenic phototrophic bacteria in the chemocline of Lake Cadagno through FISH

**Fig 4:**
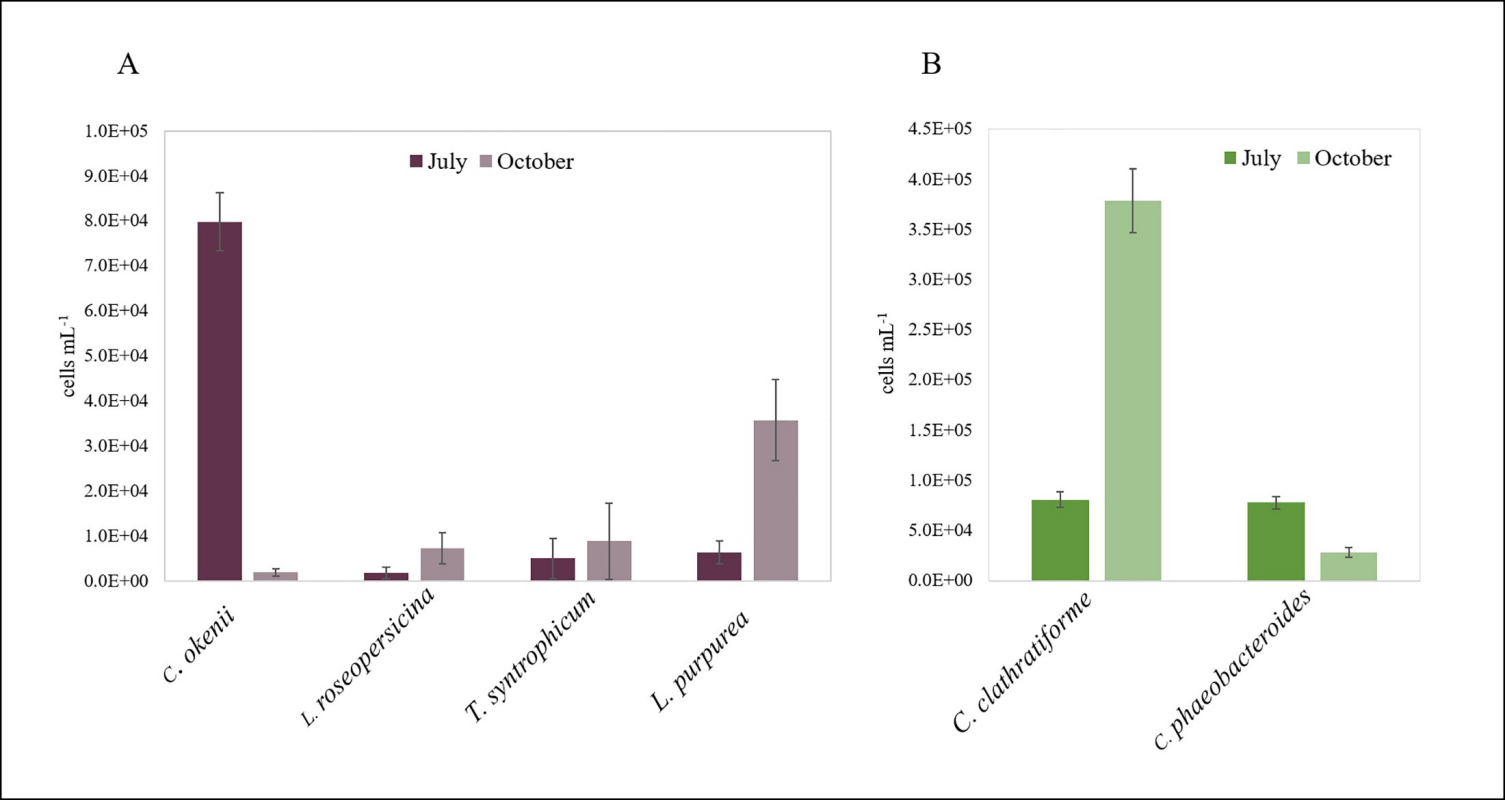
PSB and GSB FISH quantification in July and October 2016. Number of cells (cells mL^-1^) with standard error bar for (A) PSB *C*. *okenii*, *L*. *roseopersicina*, “*T*. *syntrophicum”*, *L*. *purpurea* and (B) GSB *C*. *clathratiforme*, *C*. *phaeobacteroides*. Cell concentrations were determined at maximal turbidity, corresponding to 12.2 meters depth in July and 14 meters depth in October.

Fluorescence *in situ* hybridization provided a detailed analysis of the anoxygenic phototrophic sulfur bacteria community in the chemocline for the season 2016. Total cell counts revealed concentrations up to 10^6^ cells mL^-1^. Large-celled PSB *C*. *okenii* and small-celled PSB showed different distributions in July and October (Fig 4a). *C*. *okenii* showed a drastic reduction from July (8 × 10^4^ cells mL^-1^) to October (2 × 10^3^ cells mL^-1^). Small-celled PSB “T. syntrophicum” (S453F), *Lamprocystis purpurea* (S453A), and clone 261 S453D (*Lamprocystis roseopersicina*) showed densities of 3 × 10^3^ (S453F), 1.4 × 10^3^ (S453A) and 4 × 10^3^ cells mL^-1^ (S453D) in July, respectively, and 1.4 × 10^4^ (S453F), 3.8 × 10^4^ (S453A) and 5.8 × 10^3^ cells mL^-1^ (S453D), respectively, in October. Anoxygenic GSB *C*. *clathratiforme* showed a trend to small-celled PSB reaching up to 8 × 10^4^ in July and 3.8 × 10^5^ cells mL^-1^ in October. *C*. *phaeobacteroides* showed lower reduction from July to October.

### Flow cytometric analysis of phototrophic microorganisms in the Lake Cadagno water column

**Fig 5:**
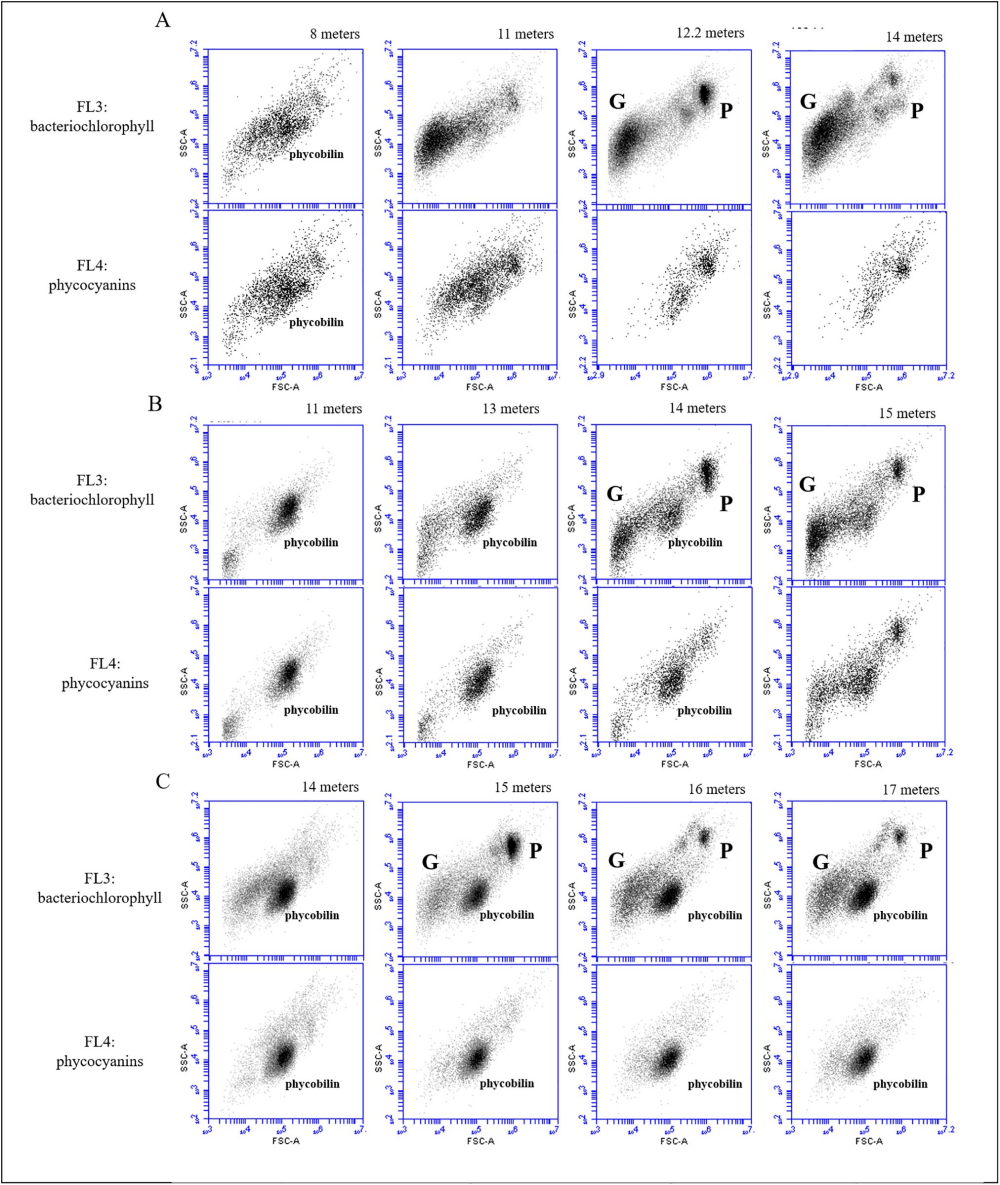
Flow cytometry detection of phototrophic populations in the oxic-anoxic transition of Lake Cadagno scatter plot SSC versus FSC for chlorophyll-pigmented cells (upper panel) and phycocyanin/phycobillin pigmented cells (lower panel). Chlorophyll and phycocyanin were used as hallmarks for phototrophic microorganisms and cyanobacteria, respectively. Threshold for pigmentation determination was set to FL3 > 1’100 and FL4 > 1’100 for chlorophyll/bacteriochlorophyll and phycocyanin/phycobillin, respectively. P: *C*. *okenii*, G: *Chlorobium* spp., phycobilin: Cyanobacteria (A) July 2016, (B) July 2017, (C) August 2017.

Flow cytometry of samples from July 2016 revealed a weak heterogeneous cyanobacteria signal (phycocyanin) at 8 m depth without the presence of an evident discernible population, i.e. at the lower part of the mixolimnion (Fig 5a, lower panel). At 11 m depth, i.e. in the upper chemocline, the cyanobacteria signal was still weak, whereas the bacteriochlorophyll signal of anoxygenic phototrophic sulfur bacteria was more pronounced. As expected, at 12.2 meters depth, large-celled PSB *Chromatium okenii* (P) and GSB *Chlorobium* spp. (G) were abundant (with 1.4 × 10^5^ and 3.11 × 10^5^ cells mL^-1^, respectively) and easily discernible through FCM (Fig 5a, upper panel). This dominance of PSB *C*. *okenii* and GSB *C*. *clathratiforme* in July 2016 was already highlighted in the previous FISH analysis (Fig 4). *C*. *okenii* showed a sharp stratification in the chemocline whereas the GSB signature was high in the chemocline and remained pronounced also at 14 m depth, i.e. in the monimolimnion. In contrast, no signal for small-celled PSB was evident in the anoxic chemocline and monimolimnion layers.

**Fig 6:**
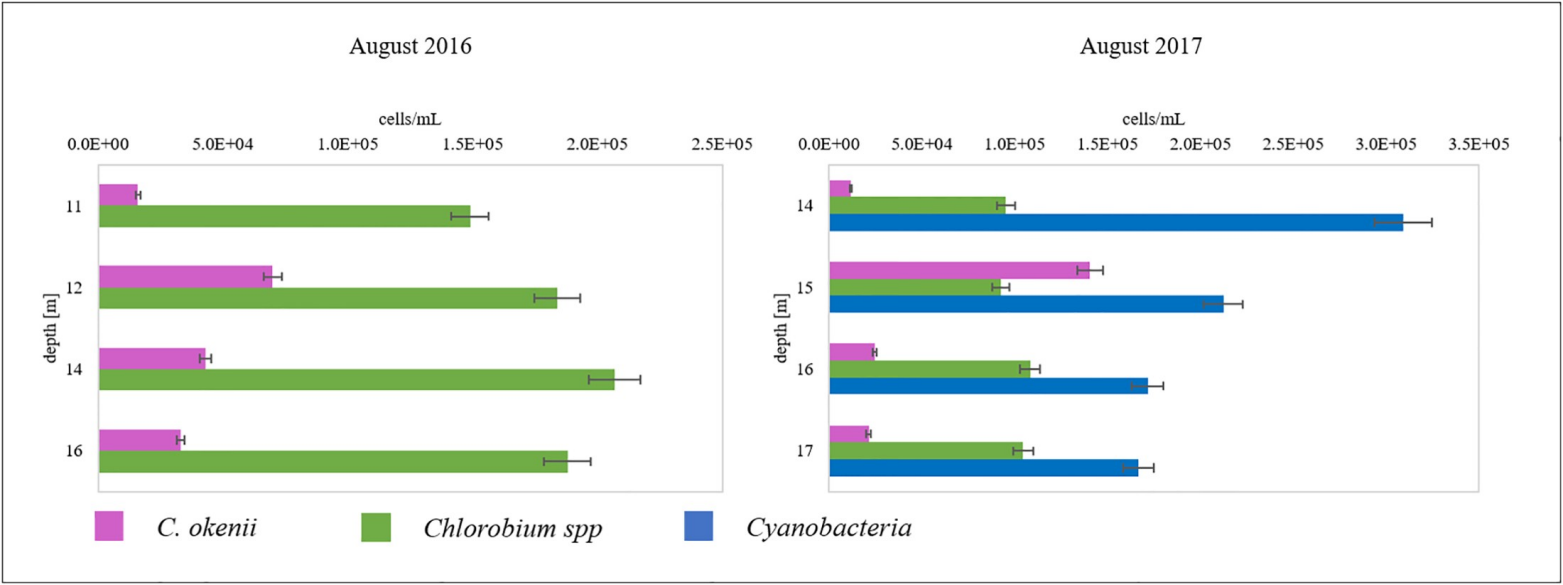
FCM determined quantification of *C*. *okenii* (P), *Chlorobium* spp. (G), and cyanobacteria (phycobilin) are reported at four depths (± 5%, maximal machine error).

The same analysis conducted one year later indicated an interesting substantial change in phototrophic microorganism composition. In July 2017, cyanobacteria (phycobilin) appeared at 11–13 m depth (1 × 10^5^ cells mL^-1^, Fig 6), i.e in the lower mixolimnion but also reached the chemocline (Fig 5b) and at 14 m depth, they co-occurred with anoxygenic phototrophic sulfur bacteria *C*. *okenii* and GSB *Chlorobium* spp. To follow the evolution of this particular cyanobacterial bloom and the consequences for the water column stratification, the FCM analysis was repeated one month later. In August 2017, the signature of this particular population of cyanobacteria increased (with maximal concentration of 3 × 10^5^ cells mL^-1^, Fig 6) when it was present throughout the water column (Fig 5c), whereas anoxygenic phototrophic sulfur bacteria showed a maximum concentration at the exceptionally deep layer of 15 meters, which was confirmed by the turbidity profile (S3 Fig).

## Discussion

Naturally stratified ecosystems such as meromictic lakes offer the unique opportunity to study how environmental gradients shape microbial communities and vice versa. In the meromictic Lake Cadagno density gradients generate distinct and stable ecosystem compartments, i.e. an oxygenic mixolimnion and an anoxygenic monimolimnion separated by a substantial chemocline [11]. The prokaryotic diversity in Lake Cadagno has been studied in the past, with emphasis on the bacterial community of the chemocline [17–20,28]. However, a complete description of bacterial richness in Lake Cadagno was lacking. In this study, we applied next-generation sequencing (454 pyrosequencing) to characterize bacterial diversity and community composition in all compartments of Lake Cadagno.

### Bacterial diversities are high in the chemocline and anoxic layer

Bacterial diversity in the chemocline and the monimolimnion of Lake Cadagno was higher than in the mixolimnion, with a Shannon-Weaver value of 5.44. Bacterial diversity in the chemocline and monimolimnion was thus higher than in other studied meromictic lakes: Lake Oigon (Shannon-Weaver value of 4.93) [42], Lake Mahoney (Shannon-Weaver value of 4.36) [43], and in Lake Pavin (Shannon-Weaver value of 3.6) [15]. As in Lake Mahoney, the greater bacterial diversity in the monimolimnion versus the mixolimnion is probably the consequence of high nutrient concentrations in the monimolimnion that promote diversity [43]. This high diversity is crucial for the maintenance of mineralization processes in the monimolimnion most of which are multistep processes that require functionally distinct taxa [44].

### Specificity of microbial communities in the different compartments of the water body

Verrucomicrobia, the most abundant phylum in the mixolimnion shows a global distribution worldwide [45] and often attains dominance in aquatic ecosystems [46,47], as in Lake Cadagno during the present study. Accordingly to its presence in the mixolimnion layer, recently reconstructed verrucomicrobial genomes described for the Opitutae classes a general heterotrophic metabolism with preference for carbohydrates, and capable of xylan, chitin, or cellulose degradation [48] Other phyla with high relative abundance in the mixolimnion of Lake Cadagno during our study comprised Alphaproteobacteria of the family Pelagibacteraceae, whereas cyanobacteria were only present at low abundance. Supporting high-throughput sequencing data, cyanobacteria were not always discernible by FCM analysis of samples from the mixolimnion/chemocline transition zone (Fig 5).

Furthermore, low relative abundance of the genus *Crenothrix* of the Gammaproteobacteria class was found in the mixolimnion. This group is usually among the major methane oxidizers in stratified lakes [49]. In accordance to these findings, it was recently shown that in Lake Cadagno methane oxidation is coupled to oxygenic photosynthesis in the anoxic water [30].

### Dominance of sulfur metabolizing bacteria in the anoxic layer

The relative abundance of Proteobacteria (Gammaproteobacteria) in the chemocline of Lake Cadagno was higher than that of other classes, corroborating past studies [19,22,31,50]. *Chromatium* accounted for 78% of total abundance (and 98% of all Gammaproteobacteria) implying its dominant role in the cycling of sulfur in Lake Cadagno. 16S rRNA gene sequencing revealed three copies in the genome of *C*. *okenii* and two copies in the genome of *Chlorobium* spp. This difference might partly explain the higher relative abundance of *C*. *okenii* inferred from pyrosequencing, however, the abundance of sequences does not necessarily correlate with the abundance of cells in a sample [51]. Evolutionary importance of polyploidy (as might be the case for *C*. *okenii*) is recognized in bacteria [52], and includes advantages in regulation of gene expression, DNA repair, and supporting large cell sizes.

In the monimolimnion of Lake Cadagno, relative abundance of GSB *Chlorobiaceae* reached 41% of the total microbial community (Fig 3). *Chlorobium* spp. are known to develop in habitats with very low light intensities, e.g in the Black Sea [53]. In deep waters of Lake Cadagno and in the absence of light, it was suggested that the most abundant GSB *C*. *clathratiforme* may obtain energy from the fermentation of polyglucose [54]. FCM analysis confirmed the presence of GSB cells in the dark monimolimnion (Figs 5 and 6). Similarly, the presence in anoxic water layer of Deltaproteobacteria Desulfobulbaceae (with 10% of relative abundance in Proteobacteria in our samples of the monimolimnion of Lake Cadagno) contribute substantially to the sulfur cycle [28].

### Seasonal dynamics of phototrophic sulfur bacteria composition and possible processes driving their evolution

The anoxygenic phototrophic sulfur bacteria community composition of Lake Cadagno chemocline was monitored since the end of past century by FISH, permitting to highlight the appearance of the novel GSB *C*. *clathratiforme* population and in general seasonal variations in phototrophic sulfur bacteria community composition [21–23]. FISH analysis in chemocline water compartment might also indicate potential seasonal dynamic pattern and possible microbial interactions.

In this study, FISH quantification of major PSB (represented by large-celled *C*. *okenii* and small-celled *Lamprocystis* and *Thiodictyon*) and GSB (*C*. *clathratiforme* and *C*. *phaeobacteroides*) revealed a seasonal evolution of the community (Fig 4). Large-celled PSB *C*. *okenii* dominated at the beginning of the vegetative period (i.e July 2016), when small-celled PSB *Lamprocystis* and *Thiodictyon* as well as GSB *C*. *clathratiforme* and *C*. *phaeobacteroides* were present at low concentrations. The drastic decrease of *C*. *okenii* observed towards the end of the vegetative period (i.e October 2016) correlated with increasing abundance of small-celled PSB and GSB *C*. *clathratiforme*. This observation is in contrast to previous temporal analyses of phototrophic sulfur bacteria distribution in the chemocline of Lake Cadagno that detected high abundance of *C*. *okenii* also in late-summer and fall [19,22,31]. It has been suggested that reduced incident light towards the end of the vegetative period favor the growth of large-celled PSB over that of small-celled PSB [55]. However, that study and a companion study [27] suggest that microbial community dynamics in Lake Cadagno might be strongly influenced by intra- and interspecific interactions.

Flagellar motility, solely represented by PSB *C*. *okenii* in Lake Cadagno, could be an important advantage in interspecific interactions, allowing a faster response to stimuli such as sulfide, oxygen or light availability variations. Recently, it was demonstrated that the motility of the *C*. *okenii* cells may cause bioconvection during the vegetative period in the chemocline of Lake Cadagno, resulting in a stratum of uniform temperature and salinity [36]. The concerted movement of large parts of C. okenii population may induce bioconvection due to accumulation of bacterial cells denser than surrounding water at the upper edge of a water volume constrained by a strong oxygen gradient [36,56]. This phenomenon could provide an advantage for *C*. *okenii* over other competing anoxygenic phototrophic sulfur bacteria by (i) vertically expanding their niche, (ii) increasing the transport of nutrients into the bioconvective layer and (iii) shuttling between vertically separated resources zones in bioconvection plumes (i.e. sulfide at the bottom and light at the top). The highest *C*. *okenii* cell concentration reported in our study (July 2016) corresponded with the seasonal maximum of mixed-layer expansion in the chemocline (~ 0.9 m) and thus probably with intensity of bioconvection [36]. Corroborating this interpretation, *C*. *okenii* population at low density measured in late-summer (October 2016) did not correlate with bioconvection and mixing observed by Sommer and colleagues [36]. Accordingly, the reduction in *C*. *okenii* concentration and the concomitant cessation of bioconvection may have reduced competition in the chemocline and created niches for small-celled PSB and GSB.

### Oxic-anoxic microbial interactions and influence on chemocline stability

The pyrosequencing approach generated a picture of microbial diversity and complexity in Lake Cadagno, highlighting specifically *Chromatium* and *Chlorobium* as key genera in the anoxic layer. Additionally FISH data indicated the particular seasonal occurrence of PSB and GSB populations.

To complete the photosynthetic water column analysis and to evidence possible microbial interactions between different lake compartments, FCM was applied as a rapid tool to detect photosynthetic microorganisms signature, as demonstrated in earlier studies [27,36,37]. Our FCM analyses of phototrophic microbial populations revealed unexpected yearly dynamics in community composition with possible effects on water column stability (Fig 5). The bloom of pycocyanin-pigmented cyanobacteria (median FSC: 105’140 ~ 2.2 μm) observed in 2017 (Fig 5b and 5c) coincided with an uncommonly drop of the chemocline at 15 m of depth, with consequent downward displacement of the anoxygenic phototrophic sulfur bacteria community to lower depths. Presence at such great depth rarely observed in past studies except in October 2000 when it was thought to be the result of a strong mixing event due to autumn storms [57]. Autotrophic picocyanobacteria were described to be present throughout the mixolimnion but scarce in the chemocline [31]. Climate warming and exceptional elevated seasonal temperature in summer 2017 might have advantaged cyanobacteria in Lake Cadagno, enhancing its vertical migration and preventing sedimentation in warmer and stratified waters increasing their resistance to grazing and favoring their buoyancy [58]. Furthermore, due to the reported higher photosynthetic efficiency [59], the extraordinary cyanobacteria proliferation event detected in our study in season 2017 might have affected the water column stability and in turn the community of anoxygenic phototrophic sulfur bacteria in the chemocline. however, at the moment we have not clear evidence for cyanobacterial oxygen production at that depth in Lake Cadagno to confirm this hypothesis. In conclusion, our study suggests that both microbial interactions between different lake compartments and within the chemocline can be a dynamic process influencing the stratification structure of Lake Cadagno water column. The identity of cyanobacteria population and the reasons for its unusual development remains still unknown and require further investigation.

## Supporting information

S1 Fig Physico-chemical and turbidity profiles of Lake Cadagno water column. Oxygen [mg L^-1^, blue line], H_2_S [mg L^-1^, orange line] (left), and turbidity profile [NTU] (right). 12 July 2017 (A), 28 July 2017 (B), 5 October 2016 (C).
(TIF)

S2 Fig CHAO1 rarefaction curves for chemocline (red), anoxic monimolimnion (blue), and oxic mixolimnion (yellow). (TIF)

S3 Fig Turbidity profile of Lake Cadagno water column taken the 28 August 2017 and showing the maximum peak at the uncommon and significant depth of 15 meters. (TIF)

## Abbreviations

FCM: flow cytometry
FISH: fluorescent in situ hybridization
GSB: green sulfur bacteria
PSB: purple sulfur bacteria
