# Supplementary figures and images for: Bacterial diversity in the water column of meromictic Lake Cadagno and evidence for seasonal dynamics
Source: PLoS One. 2018 Dec 26;13(12):e0209743. doi: 10.1371/journal.pone.0209743 (PMC6306205; doi:10.1371/journal.pone.0209743)

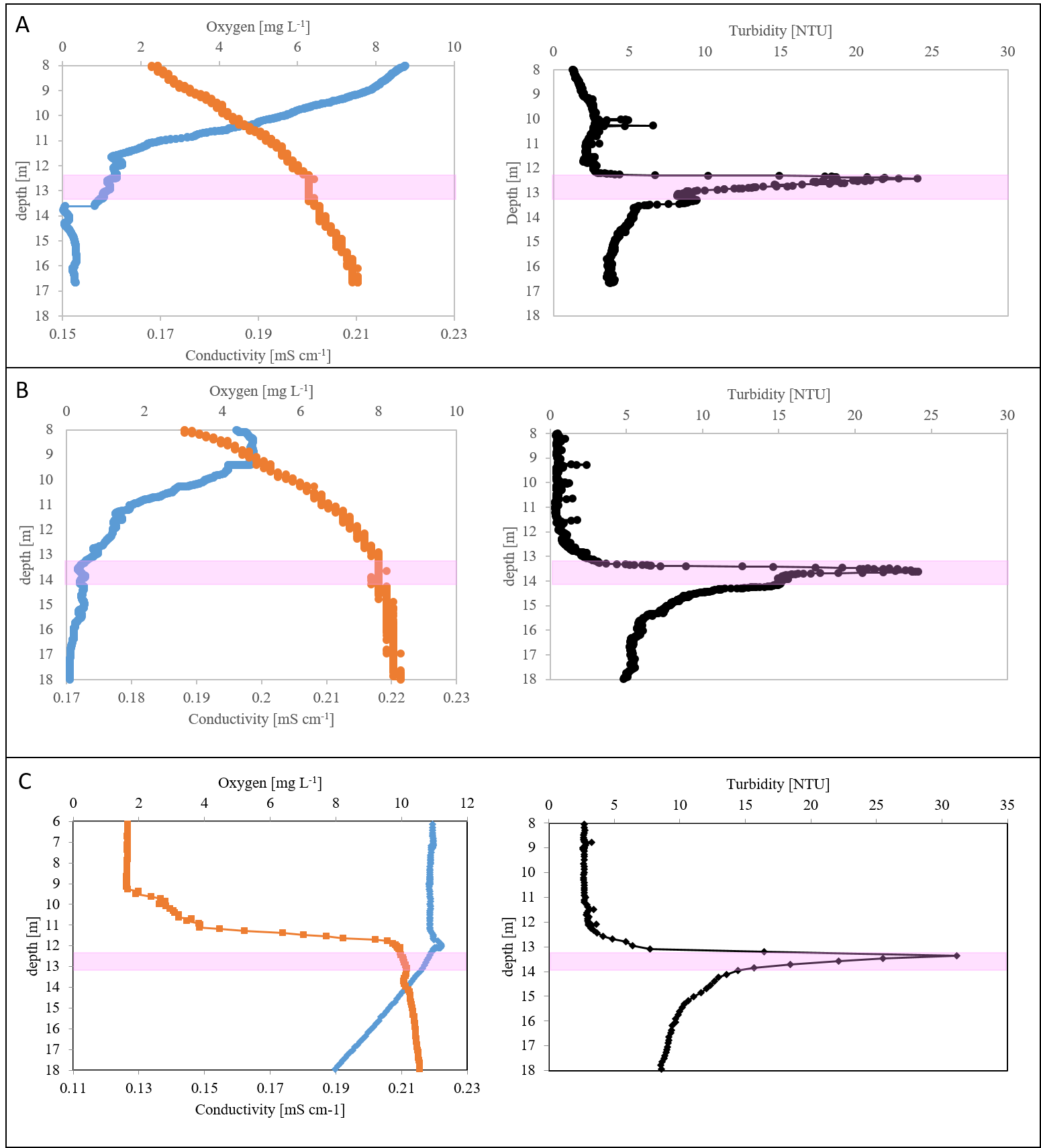

Supplement: S1 Fig — Oxygen [mg L-1, blue line], H2S [mg L-1, orange line] (left), and turbidity profile [NTU] (right). 12 July 2017 (A), 28 July 2017 (B), 5 October 2016 (C). (TIF) [file pone.0209743.s001.tif]

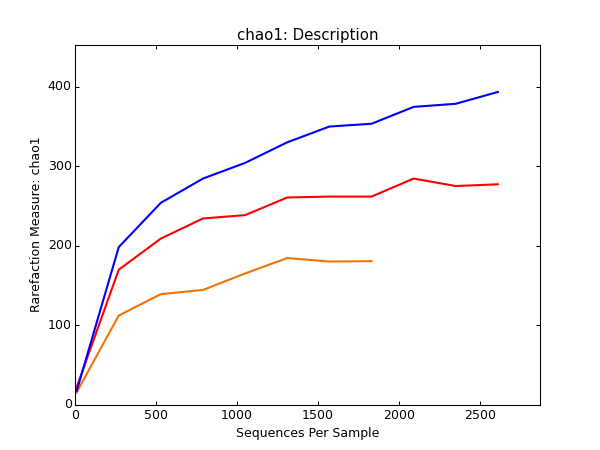

Supplement: S2 Fig — (TIF) [file pone.0209743.s002.tif]

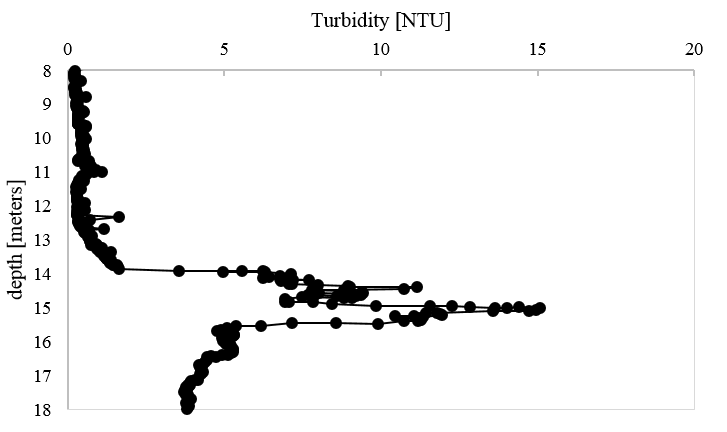

Supplement: S3 Fig — (TIF) [file pone.0209743.s003.tif]
